# Triggers of Autoimmunity: The Role of Bacterial Infections in the Extracellular Exposure of Lupus Nuclear Autoantigens

**DOI:** 10.3389/fimmu.2019.02608

**Published:** 2019-11-08

**Authors:** Connie C. Qiu, Roberto Caricchio, Stefania Gallucci

**Affiliations:** ^1^Laboratory of Dendritic Cell Biology, Department of Microbiology and Immunology, Lewis Katz School of Medicine, Temple University, Philadelphia, PA, United States; ^2^Division of Rheumatology, Department of Medicine, Lewis Katz School of Medicine, Temple University, Philadelphia, PA, United States

**Keywords:** autoantigens, autoantibodies, extracellular DNA, bacterial infections, lupus (SLE)

## Abstract

Infections are considered important environmental triggers of autoimmunity and can contribute to autoimmune disease onset and severity. Nucleic acids and the complexes that they form with proteins—including chromatin and ribonucleoproteins—are the main autoantigens in the autoimmune disease systemic lupus erythematosus (SLE). How these nuclear molecules become available to the immune system for recognition, presentation, and targeting is an area of research where complexities remain to be disentangled. In this review, we discuss how bacterial infections participate in the exposure of nuclear autoantigens to the immune system in SLE. Infections can instigate pro-inflammatory cell death programs including pyroptosis and NETosis, induce extracellular release of host nuclear autoantigens, and promote their recognition in an immunogenic context by activating the innate and adaptive immune systems. Moreover, bacterial infections can release bacterial DNA associated with other bacterial molecules, complexes that can elicit autoimmunity by acting as innate stimuli of pattern recognition receptors and activating autoreactive B cells through molecular mimicry. Recent studies have highlighted SLE disease activity-associated alterations of the gut commensals and the expansion of pathobionts that can contribute to chronic exposure to extracellular nuclear autoantigens. A novel field in the study of autoimmunity is the contribution of bacterial biofilms to the pathogenesis of autoimmunity. Biofilms are multicellular communities of bacteria that promote colonization during chronic infections. We review the very recent literature highlighting a role for bacterial biofilms, and their major components, amyloid/DNA complexes, in the generation of anti-nuclear autoantibodies and their ability to stimulate the autoreactive immune response. The best studied bacterial amyloid is curli, produced by enteric bacteria that commonly cause infections in SLE patients, including *Escherichia coli* and *Salmonella spps*. Evidence suggests that curli/DNA complexes can trigger autoimmunity by acting as danger signals, molecular mimickers, and microbial chaperones of nucleic acids.

## Introduction

Nucleic acids and the proteins that bind to nucleic acids are the main autoantigens (autoAgs) in the autoimmune disease systemic lupus erythematosus (SLE) (1). In SLE patients, autoantibodies (autoAbs) are found against lupus specific nuclear antigens, such as double-stranded DNA (dsDNA) and the Smith antigen (Sm), a non-histone nuclear RNA complex with ribonucleoprotein present in spliceosomes. Other SLE autoAbs bind different nucleic acid constituents, nucleosomes, ribosomes, and ribonucleoproteins such as Ro60 and La, and are shared with other autoimmune diseases (2). Of note, nucleic acids are not only autoAgs recognized by autoAbs in SLE, but they also represent conserved pathogen-associated molecular patterns (PAMPs) (3) of viruses and bacteria (4–8) and host nucleic acids are damage-associated molecular patterns (DAMPs) (9–14). The immune system has evolved pattern recognition receptors (PRRs) to detect the inappropriate presence of these macromolecules in the cytosolic and extracellular spaces. The compartmentalization of endogenous nucleic acids and PRRs usually prevents the inappropriate stimulation of the immune system by these potent danger signals in absence of infections (15).

The main PRRs that have been found to be involved in the pathogenesis of lupus are toll-like receptors (TLR) 7 and 9, which, respectively, recognize dsRNA and DNA rich in hypomethylated CpGs (16, 17). TLR7 and TLR9 are localized within the endosomes (18), suggesting that the origin of their ligands is extracellular and prompting the question of the source of the nucleic acids being detected. More recently, an interest has been sparked for intracellular DNA sensors, including cGAS, suggesting that nucleic acids may also be stimulating the immune system in the cytoplasm (19). Nevertheless, as autoAgs, nucleic acids have to operate in the extracellular compartment to engage B cells and autoAbs, leaving the research field wondering about source and nature of the extracellular nuclear autoAgs.

There is abundant evidence—mostly in murine models of lupus—that genetic defects in cell death and clearance of dead cells (efferocytosis) lead to release of lupus autoAgs, the combination of which can trigger autoimmunity in the right genetic background (20–23). These genetic defects, however, are rarely found in SLE patients (24), indicating the need to search for alternative causes of release of nucleic acids in the extracellular compartment.

Infections in general are thought to play a role in the development of autoimmune disease, contributing to abnormal immune responses through molecular mimicry, epitope spreading, and bystander activation (25–27). While there are multiple theories in which infection by a pathogen is thought to lead to lupus autoimmunity, a common thread that ties the many mechanisms together is that infections can lead to exposure of the immune system to nucleic acids that the host otherwise would not be exposed to (28, 29). Infections can be a dangerous and powerful source of extracellular nuclear antigens because they expose nucleic acids derived from bacteria or viruses, especially from the bacterial biofilms, which are very rich in bacterial DNA and amyloids carrying extracellular DNA (30–32). Moreover, infections can release host nucleic acids in the extracellular compartment because of the different types of cell death that can occur during infection, either as a direct cytotoxicity of the pathogen or as a consequence of normal immune responses, notably pyroptosis (33) and the extrusion of neutrophil extracellular traps (NETs) (11, 34). The interplay between infections, biofilms and cell death continues to be the focus of much discussion in the field (35–38).

**Figure 1:**
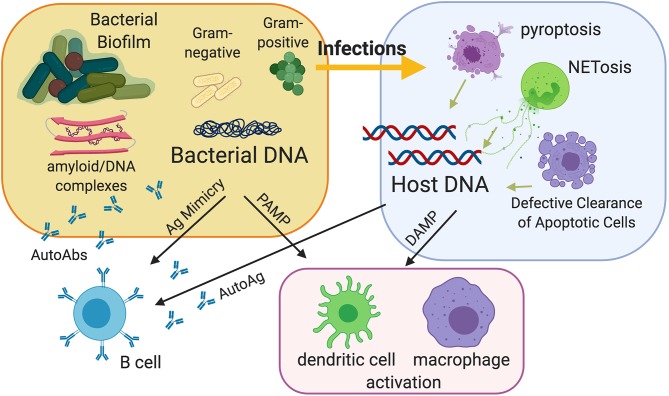
Model of the contribution of bacterial infections and bacterial biofilms to the pathogenesis of autoimmunity. Infections can be a source of extracellular nuclear antigens because they expose nucleic acids derived from bacteria and especially from bacterial biofilms, which are very rich in bacterial DNA and amyloids carrying extracellular DNA. Amyloid curli/DNA complexes can trigger autoimmunity by acting as danger signals to activate innate immunity and as molecular mimickers to activate autoreactive B cells. Moreover, infections can release host nucleic acids because of the different types of cell death that can occur during infection, notably pyroptosis and the extrusion of neutrophil extracellular traps (NETs). Defective clearance of apoptotic cells and subsequent post-apoptotic necrosis may also be a source of extracellular nucleic acids.

Circulating extracellular nucleic acids can be found in healthy individuals and were first described in 1948 (39). Their role was not associated with autoimmunity until 1966, when free DNA was found in SLE patients (40). Since then, novel techniques have shown that microorganism-derived and host-derived nucleic acids can be immunostimulatory, inducing the production of type I Interferons (I-IFNs) through both TLR-dependent and independent pathways (19, 41–45). In this review, we present findings from recent literature highlighting a role for bacterial infections and bacterial biofilms in the extracellular exposure of nuclear autoAgs, and their ability to stimulate the autoreactive immune responses in SLE (Figure 1).

## Infections are an Important Cause of Morbidity and Mortality in SLE

Infections are the leading cause of both morbidity and mortality in SLE patients, accounting for up to 55% of deaths in SLE (46–48). A large study that analyzed more than 30,000 SLE patients found that infections were a major burden, with many subsequent deaths correlated with immunosuppressive drugs and lupus nephritis (49, 50). It remains unclear whether immunosuppressive drugs and the severity of the autoimmune disease that requires such drugs predispose to infections or infections augment disease severity, or rather whether the two entities create a pathogenic vicious circle. Although lupus disease develops from an interplay between genetic and environmental factors, infectious agents have been proposed as triggers of lupus disease development due to compelling evidence of shared production of SLE-related autoAbs like anti-Sm Abs in infectious mononucleosis and cross-reactivity between SLE autoAbs and Epstein-Barr virus (EBV) proteins, which suggest the occurrence of molecular mimicry (27, 51). The most common type of infection in lupus patients is bacterial, accounting for 80% of all infections in lupus, followed by viral infections (52, 53). Viral infections are also very common in SLE patients and have been hypothesized to play a pathogenic role in SLE. A large body of literature supports a role for EBV (54, 55) and parvovirus (56–60), and most recently human papilloma virus (61, 62) has also been implicated. In this review, we focus on the perspectives of bacterial infections in lupus because of their higher incidence in SLE patients, bridging new research on biofilms and sensing of extracellular nucleic material with its implications in autoimmunity. We recommend recent reviews with excellent focus on viral infections (54, 55, 63, 64).

Among the bacterial infections, urinary tract infections (UTIs), soft tissue infections, bloodstream infections, and pneumonia are more common in SLE patients than in the general population (65, 66), either because of the immunosuppressive therapy or inherent immune abnormalities. *Streptococcus pneumoniae, Escherichia coli*, and *Staphylococcus aureus* are the most frequently associated pathogens in these infections (65, 66). Moreover, common pathogens, including *Salmonella enterica* serovar Typhimurium and *Salmonella enterica* serovar Enteritidis, appear to behave more aggressively in SLE patients; instead of causing localized gastroenteritis, *Salmonella* infection in SLE patients results in bacteremia and complications in soft tissues with high mortality rates (66–69). Additionally, SLE patients with bloodstream infections have a higher risk of developing severe flares (27, 70), making it difficult to distinguish cause and effect of the flare (27, 71–73). These results beg the question of whether infections can trigger SLE onset, or whether they are only associated with flares after the disease has started, and a definitive answer is yet to be found. Clinical studies have shown that patients with SLE who had infection-related hospitalizations suffer a profoundly increased risk of end-stage renal disease, suggesting that infections have an effect on SLE disease activity (74, 75). A study of 7,326 patients newly diagnosed with SLE showed that the occurrence of three or more infection-related hospitalizations greatly increased risk of end-stage renal disease (ESRD), indicating an effect of infections on SLE disease activity (75). The risk of infection-related hospitalizations was independently associated with ESRD following stratified analysis that adjusted for chronic kidney diseases (CKD) and other confounding factors. In the same article, the infections that had a higher hazard risk of ESRD were septicemia-bacteremia, followed by pneumonia and UTI, with soft tissue infections at the fourth place, indicating that the infections leading to ESRD were both systemic and localized to the kidney. UTIs were classified as any genitourinary infection, including pyelonephritis, UTIs and perinephric abbesses, and only patients who had infections as reason for requiring hospitalization were enrolled in the study, therefore minimizing the inclusion of iatrogenic infections such as catheter-induced ones. These data suggest a role as a promoter of lupus severity for a generalized activation of the immune system that is induced by severe bacterial infections, even when the stimulation derives from localized infection. In another study, the incidence of invasive pneumococcal infections in SLE patients was found to be 13 times higher than the incidence in the general population, an association that did not correlate with the use of immunosuppressants (76). Although the frequency of infection before lupus onset has not been thoroughly documented in the literature, some case reports suggest that it is increased, especially in pediatric lupus (77, 78), suggesting that infections accelerate SLE onset in predisposed individuals.

## Microbiome and Lupus

Recently, the symbiotic microbiota in our body have gained much attention as an influential variable conditioning human health and disease (79, 80). The gut microbiota have been subject of intense investigation because of the intriguing findings that gut dysbiosis has local and systemic effects on the immune system (81–84), but microbiota also reside beyond the gut, colonizing mucosal tissues and specific niches, from the skin to oral cavity, vagina, or the bladder, where they are expected to exercise major effects as well (85). Studies focused on lupus specifically found a reduction in species diversity in the gut microbiota that is associated with specific enteric bacteria in SLE patients (86–88) or their first-degree relatives (89) and was present in cohorts from different continents with different ethnicities (90). DNA from Enterococcus gallinarum was found in the liver of SLE patients, and colonization of autoimmune-prone mice with these bacteria induced autoantibodies and decreased survival (91). *Ruminococcus gnavus* of the *Lachnospiraceae* family is another pathobiont reported to be overrepresented in SLE gut dysbiosis and has been shown to elicit specific Ab responses correlating with anti-DNA autoAb levels, SLE activity and lupus nephritis in particular. *Ruminococcus gnavus* specific lipoglycans are proposed as novel immunodominant antigens as well as innate stimuli in SLE through the binding of TLR2 (83).

Studies exploring the role of gut microbiota on disease progression in lupus-prone mice further corroborate the importance of bacteria and infection on lupus disease development (87, 92). Consistent with findings that germ-free conditions do not influence disease outcome (93), treating lupus-prone mice with antibiotics from the time of weaning also did not impact disease activity. However, when antibiotic treatment initiation was delayed until after disease onset, SLE autoimmunity was attenuated. Lupus disease progression was thought to be attenuated by targeting Clostridial strains (i.e., *Lachnospiraceae*) found to be increased in both lupus mice and feces of human SLE patients, while allowing for beneficial commensals found in healthy individuals (i.e., *Lactobacillus* spp.) to thrive. Treatment with vancomycin, which targets Gram-positive bacteria and thus spares Lactobacilli, also reduced the translocation of LPS across the intestinal barrier, further suggesting that microbial translocation from barrier dysfunction may be an environmental trigger in SLE (94). Beside antibiotics, another variable that affects the microbiome of experimental mice and influences the severity of lupus in susceptible strains is the diet (95). An example, especially important for researchers working with mouse models of lupus, is the acidification of water that was found to decrease the levels of autoantibodies and delay the onset of nephritis in lupus-prone mice (96) while augmenting the presence of *Lactobacillus reuteri*, belonging to the phylum of Firmicutes. These results are in agreement with the lower Firmicutes/Bacteroidetes ratio that was found in SLE patients, in other inflammatory diseases, and in elderly people (86, 97–99), suggesting that bacteria belonging to the Firmicutes phylum, such as Lactobacilli ssp, are important to maintain immunological tolerance (92, 94).

## Infections as Environmental Pathogenic Factor

The association between infections and autoimmunity raises the question of why autoimmune diseases are not more common in the human population, which normally is exposed to a wide variety of bacteria and infections. Indeed, humoral autoimmunity is relatively common in the context of infections. For example, antinuclear antibodies (ANA) and rheumatoid factor (RF) are found in acute or chronic infections (100–102); the major difference is that in these circumstances, the autoantibodies are transient and do not induce a chronic defined autoimmune disease. Coupled with conflicting studies showing that the lupus-prone MRL/lpr strain of mice, which harbors a strong genetic drive for autoimmunity through the lack of the apoptotic receptor Fas, can still develop SLE-like disease in germ-free conditions, these data suggest that genetics do play an important part in disease manifestation (93). A different strain of lupus-prone mice showed instead a milder autoimmunity under germ-free conditions (103). To account for all of these findings, we envision that, in susceptible humans and mice, a genetic predisposition for immune dysfunction may increase the host exposure to nucleic acid material during infections and trigger a lymphocyte repertoire already prone to autoreactivity in the presence of specific HLA haplotypes, while in non-autoimmune-prone humans and mice, infections normally result in less prolonged exposure of a repertoire more self-tolerant to nucleic acid material. This is supported by reports of high levels of circulating endotoxin and more frequent bacteremia in SLE patients (68, 69, 89, 104).

## Exposure to Extracellular Nuclear AutoAgs in SLE

Bacterial infections can expose the immune system to nuclear material—and nucleic acids in particular—through two main processes: induction of release of host nuclear autoAgs by triggering cell death directly or as a result of the immune response against the pathogen, and the release of bacterial DNA due to bacterial death or active extrusion. The endogenous DNA, such as mitochondrial DNA (11, 105), can act as DAMP and be recognized by autoreactive B cells. Similarly, bacterial DNA, possibly associated with other bacterial molecules, can elicit autoimmunity by acting as PAMP and stimulating autoreactive B cells through molecular mimicry.

Because DNA is a major autoAg in SLE, many studies have attempted to determine whether an excess of circulating DNA may distinguish SLE patients from healthy subjects. Circulating extracellular nucleic acids were originally detected in the serum (40, 106) and then in plasma to avoid the *in vitro* artifacts due to release of cellular DNA caused by the procedure of *in vitro* coagulation (107). Initially, no differences were noted in SLE patients when compared to healthy individuals, except for SLE patients with vasculitis. SLE patients with vasculitis had higher levels of circulating DNA, suggesting that tissue damage affecting endothelia may result in the release of extracellular DNA at the site of damage (108). This was corroborated with studies showing very high levels of plasma DNA in patients who recently underwent major surgery or experienced traumatic bodily injury and, together, suggests that cell death is the source of the extracellular DNA (107, 109, 110). This concept was successfully replicated in mice, when an injection of necrotic cells induced a rapid increase of plasma DNA levels (110).

Concurrently, testing the pristane-induced model of murine lupus in mice lacking caspase-activated DNase (CAD), which results in a lack of nuclear fragmentation during caspase-dependent apoptosis, resulted in the prevention of the development of lupus by diminishing the amount of available extracellular DNA (111). Interestingly, the opposite was true when the CAD impairment was in spontaneous genetically driven lupus models, since the absence of CAD resulted in higher levels of autoAbs in triple congenic B6.Sle1,2,3 spontaneous lupus mice (112). These results suggest that in induced autoimmunity, chromatin fragmentation is essential for the presentation of nuclear autoantigens, while in mice genetically predisposed to autoimmunity the absence of nuclear modifications occurring during apoptosis promotes B cell autoreactivity, possibly by preventing the induction of self-tolerance toward DNA (112).

More recently, microparticles derived from apoptotic cells and tissue damage have been shown to be a source of these extracellular host nucleic acids and found to be present in higher numbers in the blood of SLE patients in many studies, although without full consensus (113, 114), as often seen in human studies possibly due to broad patient heterogeneity. Similar inconsistencies apply to more recent quantifications of circulating free DNA (cfDNA), which was reported to be significantly higher in SLE patients compared to controls, in correlation (115) or not (116) with high SLEDAI scores, confirming that levels of DNA, either free or bound to autoAbs or contained in microparticles, are increased in SLE patients, although the cause and pathogenic role remains to be understood.

Novel techniques allowing for plasma DNA sequencing have revealed that most circulating cell-free host DNA molecules have a size distribution that suggests a nucleosomal origin (117). These techniques are used in the clinic for non-invasive prenatal genetic testing (118) or cancer liquid biopsies, which can detect asymptomatic tumors and cancer-associated mutations (119, 120). Massive parallel sequencing revealed a spectrum of abnormalities in plasma DNA from SLE patients, including hypomethylation and fragment size shortening, abnormalities that positively correlate with levels of anti-dsDNA autoAbs and SLEDAI scores; interestingly, the abnormal DNA was bound to anti-dsDNA IgGs, suggesting that either these short sequences are specific autoAgs or they are increased because binding to Abs protected them from degradation (121). The same techniques can be used to test microbial DNA in the blood during infections and sepsis, and very recently an analytical and clinical validation of a next-generation sequencing test, which can identify and quantify microbial cell-free DNA in the plasma of patients with and without sepsis, has demonstrated the feasibility to detect in plasma the circulating free DNA of 1,250 clinically relevant bacteria, DNA viruses, fungi, and eukaryotic parasites (122). The abovementioned sequencing of plasma DNA from SLE patients used libraries of host DNA (121), leaving open the question of whether sequences of bacterial DNA are present as well, and can account for the higher levels of cfDNA found in SLE patients compared to healthy controls (115). It would be important to use these novel techniques to determine the host vs. microbial nature of circulating DNA in SLE patients.

## Induction of Pyroptosis and Other Types of Cell Death by Bacterial Infections Can Release Nuclear AutoAgs to Fuel Autoimmunity

Cell death is a natural and necessary process, and efficient recognition and clearance of products is important to avoid eliciting an immune response. Whether occurring by the programmed and regulated apoptosis or via inflammatory forms of necrosis, the accumulation of cell debris from inefficient clearance of dead bodies was proposed to cause breakdown of self-tolerance (21, 123–125). Originally, it was hypothesized that genetic defects in efferocytosis could be an underlying cause of lupus. Seminal papers reported evidence of defective phagocytosis (124, 126, 127), but genetic studies have so far identified only a few polymorphisms in genes regulating efferocytosis, or phagocytosis in general, that are linked to higher risk of developing human SLE (128, 129). Therefore, these results suggest that the defects in phagocytosis are either limited to a few patients or are not genetically determined but rather may be secondary to immunosuppressive therapies, infections or a prolonged inflammatory state.

An exception may be the specific defective clearance of nucleic acids due to loss of function of DNase 1L3. Indeed, recent studies have identified families with a high incidence of aggressive SLE and strong anti-dsDNA reactivity, in which there were children with homozygosity for a mutation in the *DNASE1L3* gene (130–133). DNASE1L3, a homologous to DNASE1, is a secreted DNase that can digest DNA in chromatin present in microparticles released from apoptotic cells (134). An SLE-associated *DNASE1L3* polymorphism (R206C) was also shown to have reduced DNase activity (135, 136). Collectively, these reports suggest that in a so far limited subset of SLE patients, the exposure of extracellular nucleic acids has a genetic cause. It remains to be determined whether the loss of function of *DNASE1L3* also affects host defense. Indeed, genetic defects in phagocytosis predispose to infections and generate a vicious circle that increases the exposure to extracellular nucleic acids. For example, the monogenic lupus due to the complement C1q deficiency is thought to be in part due to the defective clearance of immune complexes and defective uptake of dying cells, with a subsequent presence of excess extracellular host DNA (137). Nevertheless, C1q can also bind many bacterial species in an Ab-dependent and—independent manner, and C1q deficiency renders patients susceptible to bacterial infections, especially early in life (138–140). Therefore, we can speculate that an increased bacterial burden may play a pathogenic role in the development of SLE in C1q deficient patients, as in other forms of immune dysfunction, making these patients more susceptible to lupus.

It is fair to remember that mice with deficiencies in receptors necessary for efferocytosis develop SLE-like diseases including splenomegaly and glomerulonephritis and generate high levels of hallmark antinuclear antibodies (141). Mutations in BAI, MerTK, MFG-E8, Scavenger Receptor, and TIM-4 receptors involved in efferocytosis have all resulted in SLE-like disease in mice (127, 142–144). Nevertheless, caution is important when considering the direct translation of observations in mice to the human population, which is complicated by the fact that mice used in immunology are kept in specific-pathogen-free (SPF) conditions, and therefore are not exposed to the same degree of bacterial challenges that most humans see. This difference has profound consequences on the development of the immune system, as highlighted by a recent study that compared mice housed in SPF conditions with mice co-housed with pet store mice and found that the lack of pathogen experience has major effects on the cellular composition of the innate and adaptive immune systems, especially failing to elicit effector-differentiated and mucosally distributed memory T cells (145). Therefore, any conclusion on the role of genetic defects of apoptosis and efferocytosis on autoimmune outcomes warrants investigation on how natural infections may influence these murine models of autoimmunity.

Although apoptosis is most broadly recognized, a pathway of programmed cell death that is stimulated by microbial infections is pyroptosis. Pyroptosis is canonically dependent on the protease caspase 1, making this process inherently inflammatory. When caspase 1 is activated, gasdermin-D rapidly forms pores in the plasma membrane, allowing for osmotic lysis and release of inflammatory cytokines and cell contents, in contrast to the non-inflammatory apoptosis. When LPS is recognized in the cytoplasm by caspase 4 or 5 in humans (caspase 11 in mice), caspase 1-independent pyroptosis is also initiated. Both types of pyroptosis lead to the release of potent inducers of inflammasome activation and consequent inflammation. Additionally, both nuclear and mitochondrial DNA are released by pyroptotic cells (146–149).

Together with viral infections (150–152), many bacterial infections have been shown to trigger pyroptosis, and much of the tissue damage associated with such infections is caused by the induction of pyroptosis and the consequent released of DAMPs (153). Many bacterial PAMPs can trigger cytoplasmic PRRs like AIM2 and NLRPs, which are upstream of the inflammasome and the downstream caspases, and their activation leads to secretion of caspase 1-dependent cytokines IL-1b and IL-18 (154). Many bacterial models have been reported to activate the inflammasome and induce pyroptosis, from Salmonella ssp (155, 156) to Francisella novicida (157), Streptocuccus pneumoniae (158), and Listeria monocytogenes (159). Moreover, infection by Uropathogenic E. coli (UPEC) was shown to induce pyroptosis in bladder urothelial cells and release of IL-1β and IL-18 in the form of exosomes. As a consequence, mast cells migrate in the site of infection and worsen the damage to the barrier function of bladder urothelium (160).

An important inflammatory protein released by pyroptotic cells is high-mobility group box 1 (HMGB1), a nuclear DNA binding protein ubiquitously expressed in eukaryotic cells (161, 162). Circulating anti-HMGB1 antibodies are present in SLE patients and increased extracellular expression of HMGB1 is found in cutaneous lupus lesions (163, 164). *In vitro*, HMGB1, when complexed with DNA, can stimulate TLR9 and subsequent production of type I IFN by dendritic cells (165). Additionally, these HMGB1-DNA complexes can activate B cells via the receptor for advanced glycation end-products (RAGE), supporting the role of HMBG1 in promoting the formation of autoreactive B cells. Finally, our group has found that HMBG1 levels in the urine correlate with the SLEDAI and the occurrence of lupus nephritis. The highest levels were observed in class V membranous nephritis, in which they correlated with complement deposition, suggesting that the release of HMGB1 in the urine is not only due to passive excretion secondary to elevated proteinuria, but is likely linked to a mechanism inherent to class V disease (166).

Other cytokines released by pyroptosis include the caspase 1-dependent IL-1b and IL-18, both thought to play a role in promoting autoimmune disease (167–170). Moreover, many studies are reporting increased cytokines linked to pyroptosis in both human and murine SLE, contributing to lupus manifestations including nephritis. Microarray analysis of kidney tissue from SLE patients revealed an increase of inflammasome-associated transcripts (171) and low serum levels of IL-1 receptor antagonist in SLE patients suffering from renal flares suggest a pathogenic role for IL-1 in lupus nephritis (172). This enhanced pyroptosis may be due to polymorphisms in the IL-18 gene, which have been linked to SLE (173, 174) and found to lead to heightened expression of IL-18 and development of kidney disease (175, 176). These findings were further supported by the detection of heightened levels of sera and urine IL-18 in SLE patients, especially those with active lupus nephritis (177, 178). As mentioned above, bacterial infections are well-known triggers of pyroptosis (33), and common pathogens in SLE patients including E. coli and Salmonella are models of pyroptosis (155, 156, 158, 160, 179), and therefore the increased levels of this category of cell death may be due to subclinical infections causing tissue damage without generalized signs of disease manifestation. Together, these findings strongly suggest that infectious pyroptosis may play a pathogenic role in releasing host nuclear autoAgs in SLE.

NETosis is a form of cell death that specifically releases extracellular nuclear autoAgs and is triggered by bacterial infections as a weapon of host defense (180). Neutrophils are the first cells to migrate to the site of infection where they release chromatin relaxed in extracellular fibers, which can entrap Gram-positive and Gram-negative bacteria (181). NETosis is a direct antibacterial mechanism, blocking the pathogens, and it also stimulates the innate and adaptive immune response, with increased phagocytosis and production of I IFNs (125). An excess in NET formation can promote tissue damage during sepsis and many inflammatory conditions, like diabetes (182), atherosclerosis (183), and SLE (184, 185). SLE patients showed enhanced NETosis and post-translational modifications of cellular proteins, such as histone acetylation and citrullination, that can be auto-immunogenic (186, 187). NETosis also releases oxidized mitochondrial DNA, which is proinflammatory and interferogenic (11, 185), suggesting a pivotal role for NETosis in mediating the release of extracellular nuclear autoAgs in lupus. It remains to be determined whether clinical or subclinical infections are fueling NETosis, and whether genetic or environmental factors cause the increased propensity of NETting in SLE patients.

## Biofilms

Up to 80% of bacterial infections in humans are associated with biofilms (188) that bacteria build to protect themselves from environmental or immune stress (189, 190). Biofilms, a term coined by Bill Costerton in 1978 to describe a sessile, attached community of microbial cells embedded in microbe-produced extracellular matrix, was first described by Anton Von Leeuwenhoek—the pioneer of the microscope—in the 17th century (191, 192). Since then, biofilms have been defined as an aggregation of microbial cells that are firmly attached or enclosed in an extracellular matrix produced by the microbes themselves (193). Biofilms have been in the public health spotlight due to the increased recognition of their role in a number of infectious disease processes, including common infections such as UTIs, otitis media, periodontitis, and a broad spectrum of colonization of indwelling medical devices (194, 195). We are just beginning to understand the effects of biofilms on the immune system (196, 197). Very recent evidence supports a role for biofilm-forming infections in SLE pathogenesis. Indeed, Abs against periodontogenic bacteria, which produce biofilms in the oral cavity, were found to correlate with anti-dsDNA Abs and higher SLE disease activity (198), indicating a correlation between immune response to biofilm and autoreactivity. SLE patients were found to have higher prevalence of periodontal disease at younger age than healthy controls, with severe forms of periodontitis and changes in the oral microbiota characterized by decreased species diversity and higher bacterial loads, which were linked to increasing local production of pro-inflammatory cytokines (199, 200), highlighting a role for the oral microbiome in the pathogenesis of lupus.

While the primary matrix material in biofilms is extracellular polymeric substances (EPS), more than 40% of bacteria produce amyloids, proteins with a conserved quaternary β-sheet structure, which are a major structural component of the biofilm and provide the scaffold to support the biofilm tridimensional structure (32, 197, 201). The best studied bacterial amyloid is curli, produced by enteric Gram-negative bacteria that commonly cause infections in SLE patients, including *Escherichia coli* and *Salmonella spps* (197, 202). Pathological amyloids, which are generally associated with neurodegenerative disease, such as Alzheimer's or Parkinson's disease, are the result of protein misfolding and are cytotoxic for the host that produces them. In contrast, in the context of biofilm formation, bacterial amyloids such as curli are actively produced by bacteria while generating the biofilm through a finely regulated process: the main subunit protein of curli, CsgA, is synthetized by the enteric bacteria and transported to the bacterial surface, where it is polymerized into an amyloid fiber through the operons *csgBAC* and *csgDEFG* (196).

Interestingly, Robertson and Pisetsky reported in 1992 that patients with *Escherichia coli* bacteremia were positive for anti-DNA Abs and subsequently demonstrated that immunization with bacterial DNA led to or accelerated lupus-like autoimmunity in mouse models (101, 203). Our group recently reported that curli amyloids form a complex with bacterial DNA. Such binding accelerates the fibrillation of the amyloid and protects the DNA from degradation by DNases (30). Biofilms contain significant amounts of extracellular bacterial DNA, either actively extruded by live bacteria or released by bacteria upon death (204–206), some of which is bound to curli amyloids. We found that curli can fibrilize with eukaryotic DNA as well, suggesting that bacterial amyloids can not only expose the immune system to bacterial DNA, but also bind and render the host DNA immunogenic (30).

The idea that DNA complexed with a protein antigen can induce SLE-like disease has been shown by both our group and others before us (207). Di Domizio et al. showed that albumin aggregated *in vitro* into amyloid and in complex with DNA could trigger autoantibodies in a pDC dependent manner when injected in mice in presence of Complete Freund's adjuvant (208, 209), suggesting that amyloid/DNA complexes can induce autoimmunity. We discovered that injection of natural curli/DNA complexes purified from biofilms generated *in vitro* by *Salmonella* Typhimurium accelerates the development of anti-dsDNA autoAbs and anti-chromatin autoAbs in lupus-prone NZBxW/F1 mice, with the levels quickly rising by the second week of injections, without the need of any added adjuvant (30). This rapid development of autoAbs in response to curli/DNA complexes is also seen in C57BL/6 wild-type mice, suggesting a strong stimulation toward autoimmunity during infections.

Additionally, systemic infection with curli-expressing bacteria, either the commensal *E. coli* or the virulent *S*. Typhimurium, induces the production of high autoantibody titers in lupus-prone NZBxW/F1 mice. Lupus-prone mice exposed to mutant *S*. Typhimurium that cannot produce curli—and therefore cannot generate biofilms—still developed autoantibodies (30, 196), albeit at a much lower level than those infected with *Salmonella* that could produce curli. Mice exposed to curli-deficient mutant *E. coli* did not produce autoAbs at all, suggesting that exposure to curli amyloid or infection with bacteria that can make biofilms containing curli/DNA complexes stimulate the development of autoantibodies in susceptible mice (30).

Looking at the response of immune cells to curli/DNA complexes, we found that these molecules are powerful stimulators of both the innate and adaptive immune systems, inducing activation of conventional dendritic cells and macrophages *in vitro* and *in vivo*, increasing activation markers in T and B cells, and inducing the production of pro-inflammatory cytokines, like TNFα, IL-12, and IL-6, and pathogenic type I IFNs (30, 32). The mechanism of how curli/DNA elicits an autoimmune response can be explained by the ability of the amyloid to complex securely with DNA. The immunogenic curli/DNA complexes stimulate immune cells by binding to TLR2 with the β-sheet structure of curli, allowing for internalization, after which the DNA portion of the complex binds to the endosomal TLR9. Synchrotron small-angle X-ray scattering (SAXS) showed that curli organizes DNA into a columnar lattice with an inter-DNA spacing compatible with the steric size of TLR9 and maximizes TLR9 binding to DNA, leading to the amplified type I IFN response observed *in vitro* and *in vivo* (32). The role of DNA in the curli/DNA complex as a PAMP is further supported by the result that *in vitro* fibrillization of the curli monomers CsgAR4-5 polymerized into amyloids in the presence of bacterial DNA induced in dendritic cells significantly more IL-6 and IL-12 than CsgAR4-5 alone or DNA alone (30), suggesting that curli and DNA synergize to activate innate immunity.

The TLR2/TLR9 stimulation by curli/DNA complexes results in the production of type I IFNs and subsequent production of autoAbs. The autoAb production in response to curli-DNA complexes was attenuated in mice deficient for TLR2 or TLR9, suggesting that both TLR2 and TLR9 are necessary to shape the autoimmune response (32, 210). Curli/DNA was also shown to activate the inflammasome via NLRP3, extending the possible PRRs involved in their pro-inflammatory effects (210). The findings that an amyloid component of bacterial biofilms forms complexes with DNA and can potently activate a type I IFN immune response further supports the link between bacterial infections and SLE disease and highlights the important role that biofilms may play in progressing the generation of autoAbs against nucleic acids.

Curli amyloid from enteric biofilms are not the only actively produced bacterial amyloids, as homologs are found in four other phyla, i.e., *Bacteroidetes, Proteobacteria, Firmicutes*, and *Thermodesulfobacteria*, many of which are found in the human gut (211). Other bacterial species, including *Mycobacterium tuberculosis*, produce amyloids that do not share sequence homology with curli but bear the same quaternary structure and ability to strengthen the biofilm (212). Of particular relevance to autoimmunity, Gram-positive *Staphylococcus aureus* produces amyloids called phenol-soluble modulins (PSMs) (213), which fibrilize with bacterial DNA to stabilize the biofilm structure. It would be interesting to investigate the ability of PSMs and Gram-positive *Staphylococcus aureus* to stimulate autoimmunity, as we have shown for curli/DNA complexes from Gram-negative bacteria. All together, these results suggest that bacterial amyloids can act as chaperones to expose bacterial DNA to the immune system and stimulate autoimmunity in genetically predisposed individuals. Because we found that curli can bind eukaryotic DNA as well (30), we further speculate that these microbial PAMPs can also chaperone and add immunogenicity to host DNA, forming PAMP/DAMP/autoAg complexes, formidable stimulators of autoimmunity.

## Infections Trigger Autoimmunity Via Molecular Mimicry

Molecular mimicry between molecules of infectious agents and SLE-related autoAgs has been proposed as a mechanism of how SLE is triggered in a susceptible genetic background and how it leads to the breakdown of self-tolerance (214). Notably, the development of antinuclear antibodies specific for nucleic acids, arguably the hallmark of SLE, has been linked with bacterial infections in both humans and mice. We propose that curli amyloids expose bacterial DNA to autoreactive B cells and stimulate the production of anti-dsDNA autoAbs through a process of molecular mimicry. The injection of bacterial DNA induced anti-dsDNA autoAbs by the same mechanism (215), and the report that mammalian DNA did not elicit the same result can be explained by the fact that genomic mammalian DNA is not as immunogenic as bacterial DNA, especially if the latter is complexed to a TLR2 ligand like curli or another amyloid (32). Other examples of molecular mimicry were reported in SLE. Sera from human SLE patients have shown anti-dsDNA antibodies with similarity to peptides from burkholderia bacteria, and the relationship was substantiated when purified anti-dsDNA antibodies were shown to react with *Burkholderia fungorum* bacterial lysates (216). A common anti-dsDNA idiotype in humans was also found in high amounts in patients infected with *Klebsiella pneumoniae* (217). The interaction of anti-dsDNA antibodies to bacteria was also found in mice, where anti-dsDNA antibodies produced by lupus-prone mice reacted with endogenous murine flora (103, 218).

A proof-of-concept study exploring the bacterial RNA binding protein Ro60 further points to bacteria exposing homolog of nuclear autoantigens as a trigger for autoantibody production. The earliest autoantibodies in lupus are directed toward Ro60 (219, 220), and the presence of Ro60 orthologs in both lupus patients and healthy controls suggests that cross-reactivity may occur in susceptible individuals. Anti-Ro antibodies are pathogenic in lupus and are best known for leading to cardiac conduction defects and cutaneous lesions due to their trans-placental spread in neonatal lupus erythematosus (221, 222). The spontaneous development of anti-Ro60 antibodies can be induced in germ-free wild-type C57Bl/6 mice when monocolonized by a common gut commensal that produces a Ro60 ortholog (223). Within 3–5 months of monocolonization, sera are positive for anti-human Ro60 IgG. This spontaneous production of antibodies was equivalent to mice that were monocolonized by the same strain but had induced barrier inflammation and dysfunction from treatment with oral 0.1% imiquimod or 1–2% dextran sulfate sodium salt. Monocolonization with a different gut commensal does not result in the production of anti-human Ro60 IgG antibodies. Together, this model suggests that there is selective cross-reactivity between a Ro60 ortholog from commensal bacteria and human Ro60, further emphasizing how infection may play a role in triggering autoimmunity in lupus (223). This supports the concept that cross-reactivity may occur in susceptible individuals with colonization by autoantigen ortholog-producing bacteria.

Additionally, candidate antigens for the pathogenic Th cells that allow for the expansion of autoreactive B cells include those with sequences that resemble both microbial proteomes and self proteins (224, 225). The role for Th cells is well-established in SLE, and autoreactive B cells have been shown to present variable region-derived idiotype peptides on their MHC class II molecules to ideotype-specific T helper cells (226–229). Systemic autoimmune disease can be established in mice by prolonged idiotype-driven T helper cell and B cell collaboration (224, 230–235). Interestingly, an analysis of the seemingly dissimilar specificities of the T helper cells from lupus-prone mice showed a high rate of matches with microbial proteomes. Additionally, these T helper cells also developed responses toward related sequences that resembled self histones, suggesting that there is molecular mimicry between microbial peptides, idiotypes, and self proteins carrying DNA (224).

## Conclusions

In summary, the review of the recent literature presented here highlights an unmet need for studying how bacterial infections contribute to the pathogenesis of lupus and to the extracellular exposure of nuclear autoantigens in particular. Infections are a major cause of morbidity and mortality in SLE, and incomplete evidence suggests that they may accelerate SLE onset in predisposed individuals and increase disease severity in patients. Recent studies have discovered a disturbance in the microbiota profile in SLE patients and associations between pathobionts and lupus, its severity, and specific end-organ damage. These altered microbiota and repetitive infections can expose the immune system to extracellular nuclear autoAgs through host cell death and to their molecular mimics through bacterial death and extrusion of bacterial DNA. Robust experimental data supports the widely accepted working hypothesis of the complex involvement of TLRs and other PRRs in lupus pathogenesis, which is thought to promote DNA autoantibody production through activating innate and adaptive immunity. Additionally, bacterial DNA and ribonucleoproteins like Ro60 can mimic nuclear self-Ags and stimulate BCRs of autoreactive B cells in lupus autoimmunity. Furthermore, the fact that TLRs may recognize bacterial amyloid, and that bacterial biofilms contain extracellular DNA, which is bound in part to bacterial amyloid, and that they together can mimic host DNA, suggests a novel mechanism by which bacterial infections can trigger autoantibody production. The data presented here provide concrete support for bacterial infections as candidates for the extracellular exposure of lupus nuclear autoantigens, highlighting a role for bacterial biofilms in the generation of nuclear autoantigens and the stimulation of the autoreactive immune response.

## Author Contributions

CQ drafted the review and RC and SG revised it. All the authors read and approved the final version.

### Conflict of Interest

The authors declare that the research was conducted in the absence of any commercial or financial relationships that could be construed as a potential conflict of interest.
